# From rejection to the Nobel Prize: Karikó and Weissman’s pioneering work on mRNA vaccines, and the need for diversity and inclusion in translational immunology

**DOI:** 10.3389/fimmu.2023.1306025

**Published:** 2023-11-08

**Authors:** Amit Bansal

**Affiliations:** ^1^ Bergen COVID-19 Research Group and Influenza Centre, Department of Clinical Science, University of Bergen, Bergen, Norway; ^2^ Department of Infectious Diseases, University of Melbourne, at the Peter Doherty Institute for Infection and Immunity, Melbourne, VIC, Australia

**Keywords:** COVID-19, SARS-CoV-2, translational immunology, epidemiology, public health, infectious diseases, equity

## Abstract

Katalin Karikó and Drew Weissman were given the 2023 Nobel Prize in Physiology or Medicine for their findings of nucleoside base modifications that lead to the development of effective mRNA vaccines against COVID-19. This was a remarkable achievement, given that their initial manuscript was rejected by Nature and Science in 2005. The development of mRNA vaccines lagged for more than a decade for several reasons, including the lack of funding, the perceived risks of the technology, and the scepticism of many scientists. Furthermore, Karikó and Weissman’s study appeared to be technical and difficult to understand. The COVID-19 pandemic, on the other hand, has shown the importance of mRNA vaccine technology. COVID-19 mRNA vaccines have been highly effective in preventing serious illness, hospitalization, and death. The Nobel Prize for Karikó and Weissman highlights the importance of perseverance, diversity, and inclusion in translational immunology. We need to build a more inclusive scientific community, where scientists from all backgrounds are supported and their work is valued. This will result in more scientific breakthroughs and better healthcare for everyone.

## Introduction

On October 2, 2023, Katalin Karikó and Drew Weissman were awarded the Nobel Prize in Physiology or Medicine (1) “for their discoveries concerning nucleoside base modifications that enabled the development of effective mRNA vaccines against COVID-19”. This was a remarkable achievement, given that their initial manuscript on the topic was rejected by Nature and Science in 2005 (2), with the reviewers stating that the work was “not novel” and “not of interest to the broad readership.” Dr. Karikó and Weissman’s manuscript was eventually published in the journal Immunity (3) in 2005. It took more than a decade for their finding of abolished inflammatory response and enhanced protein production with base-modified mRNA (1) to be widely accepted and used in the clinic (4–7). This delay was likely due to several factors, including the lack of funding for mRNA research, the perceived risks of the technology, and the skepticism of many scientists. Additionally, Karikó and Weissman’s paper was seemingly technical and difficult to understand, even for experts in the field. In 2010, several companies were working on research and development of mRNA vaccines against Zika virus and MERS-CoV (1). In 2020, the Moderna and Pfizer/BioNTech (8) vaccines became the first mRNA vaccines to be approved for human use. The rejection of Karikó and Weissman’s paper by the Nature is a reminder of the importance of diversity and inclusion in science. The reviewers who rejected the paper may have been influenced by conscious or unconscious biases.

The COVID-19 pandemic has shown the importance of mRNA vaccine technology. mRNA vaccines have been highly effective (9) in preventing serious illness, hospitalization, and death from COVID-19. However, the delay in the development of mRNA vaccines has cost millions of lives. There are several reasons why it took more than a decade for mRNA technology to be in the clinics (1, 8). First, mRNA is a very unstable molecule, and it is difficult to deliver it to cells without it being degraded. Second, early mRNA vaccines were often associated with severe side effects, such as inflammation and fever. However, Karikó and Weissman developed a base-modified mRNA (1) that was more stable and less likely to cause reactogenicity.

## The role of other COVID-19 vaccines, particularly from non-Anglo-Saxon countries

**Figure 1:**
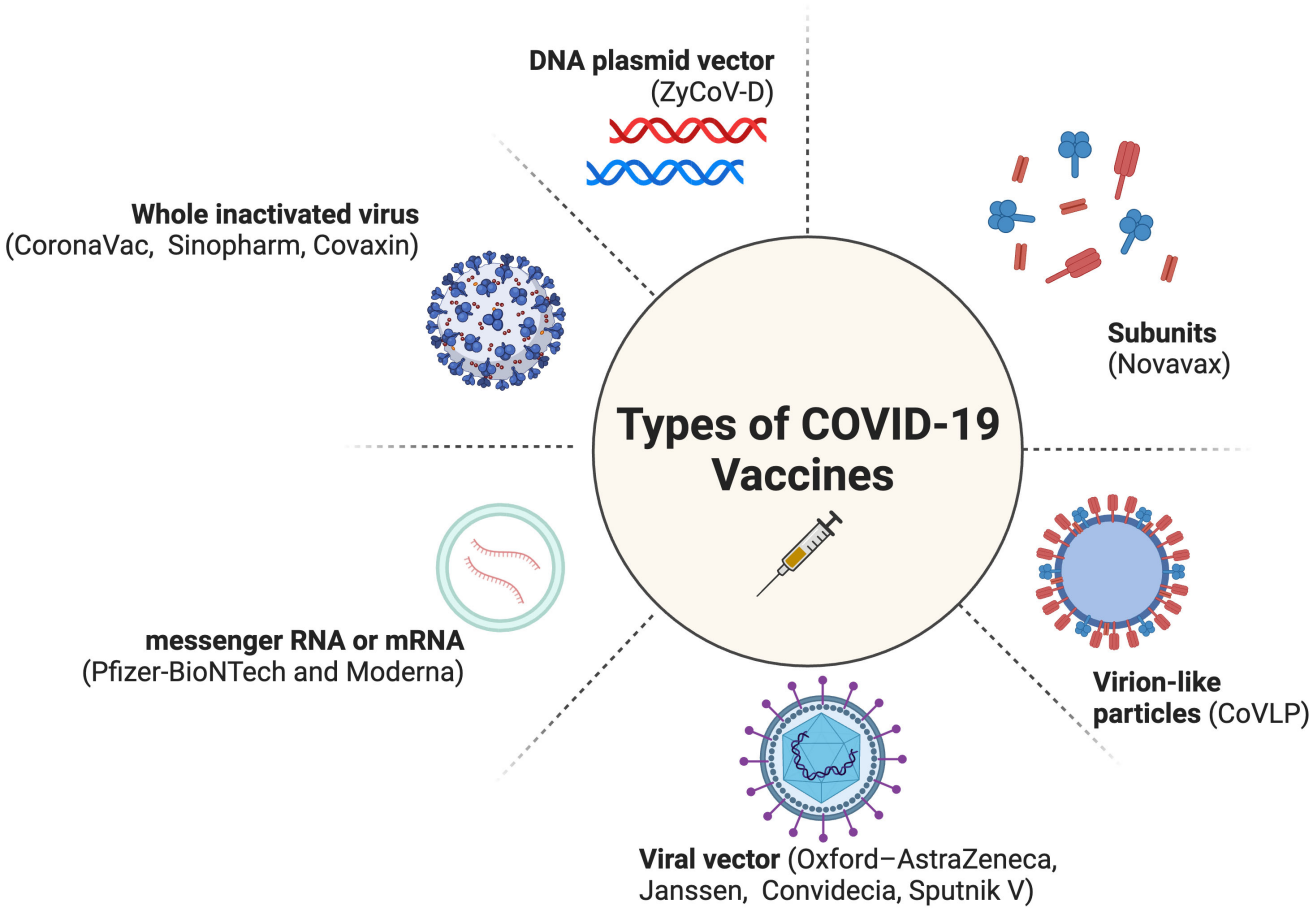
Types of COVID-19 Vaccines (15, 16). Created with BioRender.com (2023).

COVID-19 vaccines from non-Anglo-Saxon countries, such as India (10–12), China (13), and Russia (14), have not received due credit for their role in the pandemic as the Moderna and Pfizer-BioNTech vaccines (**Figure 1**). This is even though these vaccines have been helpful in saving tens of millions of people around the world (10, 17). This highlights stark disparities, specifically a disproportionate amount of global appreciation and public trust in the research derived from Western countries as opposed to other parts of the world. The lack of recognition for these vaccines could be due to factors such as racism, chauvinism, and geopolitical rivalry; however, essentially there has been some concern about the efficacy of these vaccines. Fortunately, recent studies have shown that they are effective in preventing serious illness and death from COVID-19 (10–14, 18). It is important to recognize the contributions of all scientists to the global fight against COVID-19, regardless of their nationality.

## Promoting diversity and inclusion, so that lab research does not have to wait for decades to reach clinics

We need to do more to promote diversity and inclusion in science. “Hopefully, this prize will inspire women and immigrants and all of the young ones to persevere and be resilient. That’s what I hope,” says Karikó (19). This will help to ensure that all scientists can have their work considered fairly, regardless of their race, gender, or nationality. We also need to support research on new technologies, even if they are seen as risky or unconventional. Only then can we ensure that the fruits of scientific discovery are available to everyone. Karikó and Weissman, both pioneers in mRNA research, have had very different careers. Karikó languished permanent academic post at the University of Penn likely due to gender discrimination and lack of funding, while Weissman became a successful professor and “faculty quality”, despite studying the same biology. This highlights the importance of supporting women in science and addressing gender discrimination. Furthermore, industry provided more financial support for Karikó’s work on mRNA vaccines than academia. Despite the obstacles she faced, Karikó persevered and made ground-breaking contributions, leading to the development of life-saving mRNA vaccines. We can learn from her story to persevere in the face of challenges and never give up on our dreams. We should not be overly concerned about rejection by top medical journals. Our scholarly work will open new doors and lead to new opportunities. Academic feedback, which is unfortunately usually unpaid, can refine and enhance scholarly work.

**Figure 2:**
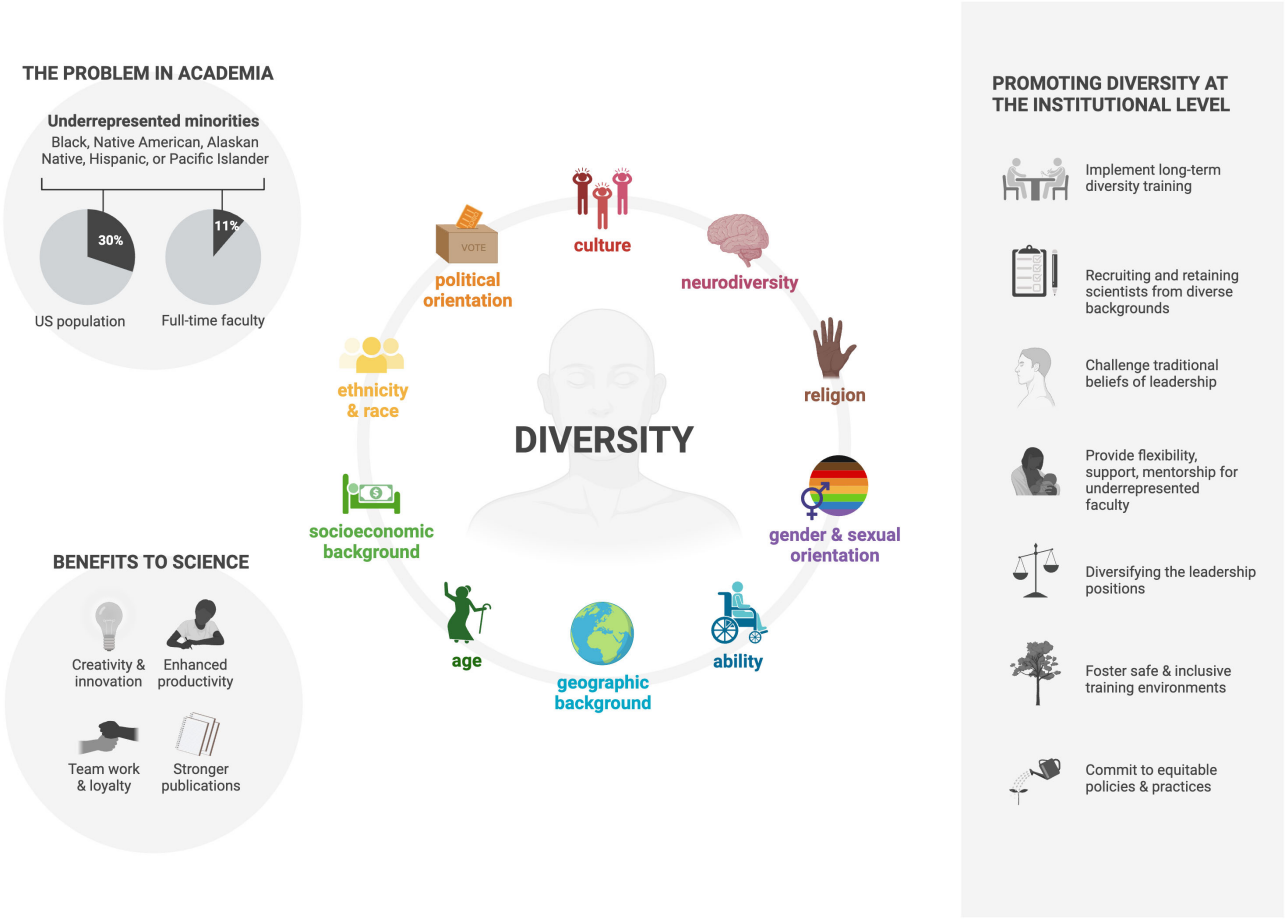
Increasing Diversity in the Immunology Research Community (20, 21). Created with BioRender.com (2023).

There are several specific things (**Figure 2**) that can be done to promote diversity and inclusion in science, including:

• Recruiting and retaining scientists from diverse backgrounds: This can be done by providing scholarships and fellowships to scientists from underrepresented groups, and by creating a welcoming and inclusive environment for all scientists.
• Diversifying the leadership of scientific institutions: Crediting minoritized medical faculty expertise and lived experience will help to ensure that all voices are heard and that all perspectives are considered (22). Unfortunately, in several leading universities, you will hardly find under-represented racial and ethnic minorities in leadership roles. Likewise, there is no self-identified Black recipient of the Nobel Prize in Physiology or Medicine (23).
• Funding research on a wide range of topics, including those that are important to underserved communities. This will help to ensure that all scientists can make a difference.
• Educating the public about the importance of diversity and inclusion in translational medicine. This will help to create a more supportive environment for scientists from all backgrounds.
• By taking these steps, we can create a more diverse and inclusive scientific community that is better equipped to solve the challenges of the 21^st^ century.

## Conclusion

The work experience of Karikó and Weissman is a reminder of the importance of perseverance, diversity, and inclusion. We need to create a more inclusive scientific community, where scientists from all backgrounds are supported and their work is given due consideration. This will lead to more scientific breakthroughs and better healthcare for everyone. We also need to recognize the contributions of all scientists to the global fight against COVID-19, regardless of their nationality.

## Data availability statement

The original contributions presented in the study are included in the article. Further inquiries can be directed to the corresponding author.

## Author contributions

AB: Conceptualization, Funding acquisition, Visualization, Writing - original draft, Writing - review & editing.
